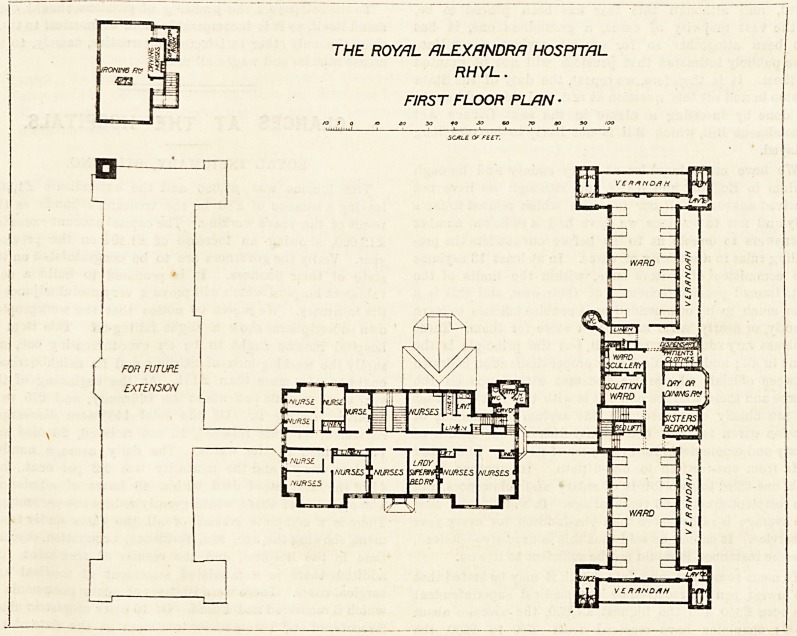# Alexandra Hospital for Children, Rhyl

**Published:** 1904-01-16

**Authors:** 


### HOSPITAL ADMINISTRATION.

### CONSTRUCTION AND ECONOMICS.

### ALEXANDRA HOSPITAL FOR CHILDREN, RHYL.

THIS hospital consists, at present, of four blocks. One is given up to administration purposes, and another contains the wards. These are joined by a corridor. The third contains the laundry, and the fourth is for the engine and boilers. With a view to future extension the latter much resembles one wing of the ward block. Generally speaking it is desirable in a hospital of this size to incorporate the laundry with the boiler-house block and to place them as far as possible from the wards, having regard, however, to the distance which steam will travel without much loss to its heating power when it has to be used for cooking or warming. In the plans supplied to us no kitchen department is shown.

The site seems to have been well chosen, as it commands at least three roads. The patients' entrance is approached from Alexandra Road, and that of the administration block is from another road not named on the plan. The administration block has on its entrance side a dining-room, visitor's-room, sisters'-room, and nurses'-room. Beyond these a corridor runs from which open the lady superintendent's room, the secretary's office, the staircase, the chapel, another staircase, and the sanitary arrangements. No committee-room is shown. The corridor has been expanded and made octagonal at both ends, by which means more light is obtained, and the appearance improved of a part of a hospital too often far from pleasing. Excepting part of the chapel this block is carried up to a first floor, and good accommodation is provided in it for the nursing staff.

The patients' block is put on at right angles to the connecting corridor, and it is divided into two wards, these having between them the staircase, with bed-lifts, sister's-room, day-room, scullery, linen-room, clothing store, isolation-room, and (what we do not remember having before seen in this position) the dispensary. Each ward contains 12 beds, and excepting the end beds each bed has a window on both sides of it. The sanitary annexes project from the ends of the wards and are properly cut off by ventilating lobbies. Verandahs are placed at the ends and sides of the wards. From the plan it will be seen that neither ward is overlooked by the sister's-room, which is a mistake; and the isolation ward is too closely incorporated with the block to be of much use for isolation purposes. Moreover, its position prevents cross-ventilation other than it may obtain from a fanlight over the door, and even then it would ventilate into the main corridor leading to the ward. The first floor is a replica of the ground floor.

Presumably with the object of economising ground space, the laundry block is of two stories; and the ironing-room and drying-closets are placed on the first floor, lifts being used to get the clothes up and down.

In general conception the design of this hospital is decidedly good, and when the extensions are built it will present an appearance of compactness, yet with plenty of air space around the blocks, and it ought to work harmoniously. We think it therefore the more to be regretted that the points we have noted adversely should exist.

The architects are Messrs. Waterhouse & Son, of New Cavendish Street, London.

## Figures and Tables

**Figure f1:**
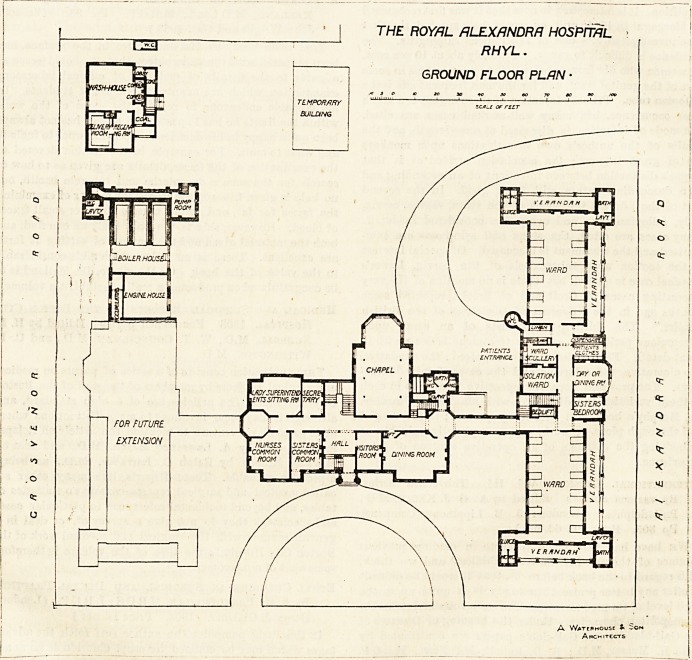


**Figure f2:**